# Supplementary material for: “I think they are infected because of their ignorance and lack of responsibility”: A mixed-methods study on HIV-related stigma in the healthcare system in Kazakhstan
Source: PLoS One. 2025 Sep 2;20(9):e0331201. doi: 10.1371/journal.pone.0331201 (PMC12404490; doi:10.1371/journal.pone.0331201)
Supplement: S1 File — (DOCX) [file pone.0331201.s001.docx]

**HIV-Related Stigma in Healthcare Settings: Detailed Guide for Conducting In-Depth Interviews (Semi-Structured)**

**Introduction**

- Introduction of the interviewer, brief overview of the study objectives and goals. Explanation of the training timeline.
- Explanation of the interview process and participation options in the study, responding to questions raised during the interview methods.
- Obtaining informed consent.

**Beginning of the Interview**

**Questions**

1. Professional information.
2. Please tell us a little about your work and what responsibilities you have here.
3. What is your job title?
4. How many years/months have you worked at this healthcare institution and in your field in general?
5. What is your experience working with HIV-positive patients?

**Experience of working with people living with HIV (PLHIV)**

General question: What comes to your mind first when you think about HIV and the risk of HIV transmission in healthcare settings?

1. What risks can you imagine when providing care to HIV-positive patients?
2. What are your regular precautions during medical procedures with HIV-positive patients?
   **PROBE:** How can these precautions be improved?
3. What do you think about training courses on "HIV and care for HIV-positive patients"?
   **PROBE:** What are the most important aspects of care for HIV-positive patients that should be included in these courses, and why? How do you think these courses could be made more effective? Any additional comments or suggestions?
4. Some previous studies/practices show refusals of medical care for HIV-positive patients in healthcare settings. What do you think about this?
   **PROBE:** What do you think about the rights of patients and medical staff regarding patient care? Can you recall any problematic situations between patients and healthcare staff in your institution?
5. What do you do differently when treating HIV-positive patients compared to HIV-negative patients? Why? Why not?
6. What do you think about the adequacy of precautionary measures to prevent HIV transmission in your healthcare institution?
   **PROBE:** Materials for hand hygiene, single-use items, personal protective equipment (PPE), injury prevention rules, and disposal of medical waste, etc.?
7. What do you think about the policies and protocols for providing care to HIV-positive patients in your healthcare institution?
   **PROBE:** Can you describe the regular practice of caring for HIV-positive patients in your institution, including testing, disclosure of HIV status, etc.? Can you recall any experiences or issues raised regarding these policies among your colleagues?
8. How would you describe your colleagues' attitudes toward HIV-positive patients in your healthcare institution?
   **PROBE:** Can you suggest any ways to improve care for HIV-positive patients? Do you have any ideas on improving conditions for better interaction/working with such patients?

**Attitudes toward PLHIV**

General question: What comes to your mind first when you think about people living with HIV?

1. What do you think about people who acquire HIV through sexual intercourse?
2. What do you think about people who acquire HIV through injecting illicit drugs?
3. How would you feel if someone close to you (a friend or family member) contracted HIV?
4. What is your general attitude toward sex workers?
   **PROBES:** How would you feel if someone close to you (a family member or friend) engaged in sex work?
5. What is your general attitude toward people who inject drugs (PWID)?
   **PROBES:** How would you feel if someone close to you (a family member or friend) was a PWID?
6. What do you think about non-traditional sexual behaviour?
7. What is your personal attitude toward non-traditional sexual orientations and behaviours related to this topic (both globally and locally)?
8. Some healthcare workers prefer not to provide medical care to HIV-positive patients. What do you think are the reasons for this? What about your own practice—how would you feel/what do you think about it?
9. What do you think about people living with HIV who would like to start a family and have children, if they wish to do so?

**Knowledge about HIV Transmission**

1. How would you rate your knowledge about HIV?
2. How would you rate your colleagues' knowledge about HIV? What topics are still missing in medical education (e.g., continuing education courses), etc.?

**Conclusion of the Interview**

What advice would you give for managing HIV-positive patients in your healthcare institution?

Is there anything else you would like to add that we did not ask about?

**Thank you for your participation!**
