# Supplementary material for: “I think they are infected because of their ignorance and lack of responsibility”: A mixed-methods study on HIV-related stigma in the healthcare system in Kazakhstan
Source: PLoS One. 2025 Sep 2;20(9):e0331201. doi: 10.1371/journal.pone.0331201 (PMC12404490; doi:10.1371/journal.pone.0331201)
Supplement: Attachment — Submitted filename: Response_to_reviewers.docx [file pone.0331201.s004.docx]

Manuscript PONE-D-24-31521

Response to Reviewers

Dear Reviewers and the Editorial Team,

We would like to express our sincere gratitude for your time and effort in reviewing our manuscript titled *"I think they are infected because of their ignorance and lack of responsibility": A Mixed-Methods Study on HIV-Related Stigma in the Healthcare System in Kazakhstan*. Your thoughtful and thorough comments have been invaluable in improving the quality of our work.

We truly appreciate the detailed and constructive feedback you provided. Your insights have significantly contributed to enhancing the clarity of the manuscript. In response to your suggestions and comments, we have made the necessary revisions and ensured that all edits are clearly marked for your convenience. Specifically, we have used the review mode with track changes on the manuscript and highlighted any amendments made (in yellow). In the current document, we have provided our responses in blue font and included the new line numbers for your convenience.

Once again, thank you for your time, dedication, and expertise. We highly value your contribution to this work and look forward to any further comments you may have.

Sincerely,

Corresponding author,

Balnur Iskakova, PhD

School of Public Health

Asfendiyarov Kazakh National Medical University

**Journal Requirements:**

1. When submitting your revision, we need you to address these additional requirements.

We have revised and corrected the manuscript to fully comply with Plos One's style requirements, including the specified file naming conventions.

2. Thank you for stating the following in the Acknowledgments Section of your manuscript: 
We note that you have provided funding information that is not currently declared in your Funding Statement. However, funding information should not appear in the Acknowledgments section or other areas of your manuscript. We will only publish funding information present in the Funding Statement section of the online submission form. 
Please remove any funding-related text from the manuscript and let us know how you would like to update your Funding Statement. Currently, your Funding Statement reads as follows: 
The author(s) received no specific funding for this work.

Please include your amended statements within your cover letter; we will change the online submission form on your behalf.

Thank you for this comment. This research was conducted as part of the first author's PhD program at Asfedniyarov Kazakh National Medical University without specific funding. However, the first author benefited from training provided by the Fogarty International Center of the National Institutes of Health under Award Number D43TW010046 during her PhD studies. The acknowledgment section has been revised now as follows:

*“We appreciate the support provided by the Fogarty International Center of the National Institutes of Health under Award Number D43TW010046 for offering biostatistics and epidemiology courses on HIV-related research during the first author’s PhD program (Non- certificate degree at SUNY Albany).”*

3. We note that this data set consists of interview transcripts. Can you please confirm that all participants gave consent for interview transcript to be published?

If they DID provide consent for these transcripts to be published, please also confirm that the transcripts do not contain any potentially identifying information (or let us know if the participants consented to having their personal details published and made publicly available). We consider the following details to be identifying information:
- Names, nicknames, and initials
- Age more specific than round numbers
- GPS coordinates, physical addresses, IP addresses, email addresses
- Information in small sample sizes (e.g. 40 students from X class in X year at X university)
- Specific dates (e.g. visit dates, interview dates)
- ID numbers

Yes, we obtained explicit informed consent from the participants for the quotes from the transcripts to be published. This consent is documented and can be found in the interview guide, which we are attaching in three languages as part of our revised submission.

Regarding potentially identifying information, we ensured that the transcripts do not contain any such details. Specifically, we only collected participants' age in round numbers, information about their position or current role, years of work experience, and whether they have ever provided care to people living with HIV (PLHIV). No identifying information, such as names, specific ages, addresses, or other details listed in your guidelines, is included in the transcripts.

**Comments to the Author**
**Reviewer #1**
General comments:

• Insert page numbers throughout the manuscript
Thank you for your suggestion. We have now inserted page numbers throughout the manuscript, as requested.

**Abstract**:
• (Line 33/line 34 under Methods) change “bivariate associations” into bivariate analysis to refer to the statistical analysis of the relationship between two variables.

Thank you for your suggestion. We have made the requested change and replaced "bivariate associations" with "bivariate analysis" on line 34.

• (Line 34/line35 under methods) it would be better to say, “models were performed” instead of “models were used” for a more precise and formal tone.

Thank you for your suggestion. We have now made the requested change from “models were used” to “models were performed” on line 35.

• (Line 40-42/line 42-44 under results) This statistical reporting “(AOR=0.25; 95%CI=0.09,0.67; p=0.006)”, must be formatted in full at first appearance to improve clarity as follows (AOR [Adjusted Odds Ratio] =0.25; 95%CI [95% Confidence Interval] =0.09, 0.6; p=0.006).
Thank you for your suggestion. We have now made the requested change as follows:

*“Logistic regression analysis indicated that longer years of work in healthcare were protective against stigmatizing opinions (Adjusted Odds Ratio (AOR) =0.25; 95% Confidence Interval (95%CI)=0.09,0.67; p=0.006), while not having seen an HIV-positive patient in the last 12 months was associated with higher stigma (AOR=3.31; 95%CI=1.73, 6.31; p<0.001)”*

**Introduction**:

• (Line 61/line 61-64) Reformat and restate the following “…. estimates (2020)……” by simply removing parentheses as follows “…..estimates in 2020…”.

Thank you for the suggestion. We have revised and updated the prevalence statistics to reflect the most current data on line 61-65:

*“There were 40,000 PLHIV in Kazakhstan by the latest estimates in 2023, and the highest prevalence of the infection is documented in several regions including Pavlodar, Karaganda, Almaty, North Kazakhstan, Kostanay, and East Kazakhstan [1,4,5].”*

• (Line 65 -68/line 66-69) I recommend that the authors report on the achievement of the 2020 90-90-90 targets alongside the progress toward the new, ambitious 95-95-95 targets in the Kazakhstan context for viral suppression, set by UNAIDS for 2025.

Thank you for the recommendation. We have now included both the achievement of the 2020 90-90-90 targets and progress toward the 95-95-95 targets for viral suppression in Kazakhstan, as recommended on line 66-69.

*“Although Kazakhstan has made progress, it has not yet achieved the 90-90-90 targets. Thus, in order for the country to reach the more ambitious 95-95-95 goals by 2025, substantial efforts are required, particularly in addressing stigma and discrimination”*

• (Line 67-68/line 72-76) The following sentence must be cited, and I strongly recommend the citations of recent statistics “Notably, the number of new HIV infections in Kazakhstan increased by 68 39% in the years of 2010-2019.”

Thank you for your recommendation. We have now cited the relevant source and updated the prevalence statistics with the latest data. Additionally, we have revised the reference accordingly.

*“Notably, HIV incidence in Kazakhstan surged by 88% from 2010 to 2021, making it the seventh highest increase globally, while the number of people living with HIV more than doubled [7]. The prevalence rates among key affected populations such as people who inject drugs (PWID), men who have sex with men (MSM), prisoners, and sex workers (SWs) were 7.60%, 8.80%, 4.20%, and 1.50%, respectively in 2023 [8].”*

• (Line 78-79/line 80-82) This sentence warrants citation “The term ‘stigma’ is associated with “exclusion”, “rejection”, or “blame” of one due to holding a particular condition which is a subject for social judgment.”

Thank you for your recommendation. We have now cited the relevant source.

*“HIV is one of the world’s highly stigmatized conditions. The term ‘stigma’ is associated with “exclusion”, “rejection”, or “blame” due to holding a particular condition, which is a subject for social judgment [9].”*

9. Florom-Smith AL, De Santis JP. Exploring the concept of HIV-related stigma. Nursing Forum. 2012;47(3):153–65.

• (Line 77-79/line 82-85) Citation is required for this statement: “Stigma associated with HIV/AIDS can also be self-internalized by PLHIV, meaning that the general negative reactions to HIV from others may result in “internalized” stigmatization among PLHIV.”
Thank you for your recommendation. We have now cited the relevant source.

*“Stigma associated with HIV/AIDS can also be internalized by PLHIV, meaning that the general negative reactions to HIV from others may result in “internalized” stigmatization among PLHIV [10].”*

• (Line 74-84/line 79-90) The multifaceted concept of stigma has been conceptualized, but this has primarily focused on self-stigma and enacted stigma. However, public stigma and perceived stigma, which may be relevant in the context of this study, have been largely overlooked.

We appreciate the reviewer’s thoughtful comments on the multifaceted concept of stigma. While various terms are used to conceptualize stigma, in this study, we chose to focus specifically on anticipated, internalized, and enacted stigma. Our intention was to highlight the primary forms of internal and external stigma relevant to our findings without delving too deeply into the multiple subtypes. However, we recognize the importance of public and perceived stigma, and we note that perceived stigma closely aligns with anticipated stigma, while public stigma parallels the enacted stigma we addressed. We are open to incorporating a brief reflection on these aspects in the Discussion section. If the reviewer has specific resources or references to recommend, we would gladly consider including them to enrich our manuscript further.

• (Line 86/line 88) Address the typo “feelings wile enacted stigma” write while for clarity

Thank you for pointing out the typo errors. We have corrected and it now reads as *“Internalized stigma may lead to low self-esteem, self-imposed withdrawal, and even suicidal feelings, while enacted stigma may exacerbate such feelings, resulting in an unwillingness to engage with others [10-12].”*

• (Line 87-88/line 89-90) This assertion needs citation “Fear of disclosure and shame caused by HIV-related stigma may keep PLHIV from getting the care needed in time.”

Thank you for your recommendation. We have now cited the relevant source on line 88-89.

*“Fear of disclosure and shame caused by HIV-related stigma has been shown to keep PLHIV from getting the care needed in time [11].”*

11. Dessie ZG, Zewotir T. HIV-related stigma and associated factors: a systematic review and meta-analysis. Frontiers in Public Health. 2024 Jul 23;12:1356430. .

• (Line 87/line 93) Rephrase the term caregivers “For example, caregivers may” to healthcare providers.
Thank you for your suggestion. We have now rephrased “the caregivers” to “healthcare providers” on line 93.

• (Line 94/line 95-97) specify the context (country or region) in which this study was conducted “Previous research conducted in Europe…”

We realized that the cited European paper referenced international research. Therefore, we updated the reference and paraphrased the sentence in the manuscript. The revised sentence now reads:

***“Studies on HIV-related stigma conducted in China and Vietnam suggest that healthcare staff may adopt specific attitudes and behaviors to align with their peers and gain social acceptance [13,14].”***

• (line 93-94/line 99-101) Authors ought to clarify what they mean with “regular procedure” given that this phrase carries significant ethical implications particularly with regards to patient confidentiality and ethical responsibility of Healthcare Providers. This point must be elaborated.

We agree with this point and have revised the phrasing accordingly. The term “regular procedure” has been clarified to better reflect the ethical concerns surrounding patient confidentiality and the responsibility of healthcare providers.

*“Although some healthcare providers in resource-limited settings may prefer to visibly mark the files PLHIV for perceived safety reasons, this practice can lead to breaches of patient confidentiality and reinforce stigma [14].”*

• (Line 111/line 113) Revise this grammatical error “exploratory study is aimed to….” to “This exploratory study aims to assess….”

Thank you for your suggestion. We have now made the requested grammatical change and now it reads as *“This exploratory study aimed to assess HIV-related stigma in Kazakhstani primary healthcare settings and the factors leading to stigmatization of PLHIV using a mixed methods approach”*

**Methods**:

• (Line 109/line 116) This section should be written as “Material and Methods”
Thank you for your suggestion. We have now changed the heading to “Materials and methods”

• (Line 116 -118/line 123-129) Under the subheading of “Population and Sampling”, it is essential to include the sample size formula along with its corresponding parameters defined to demonstrate how the total sample size of 380 was determined. This should clearly indicate whether the figure represents a minimum or maximum sample size. This transparency will enhance the understanding of the sampling process and ensure the validity of the research findings.

Thank you for your comment. The sample size for this study was determined based on the most recent data, which indicated that approximately 13,267 healthcare professionals, including doctors and nursing staff, were employed in medical institutions across Almaty in 2017. The required sample size was calculated using a population proportion formula for infinite populations:

n0=E2Z2⋅P⋅(1−P)

2

This initial calculation yielded a sample size of n0=384. To account for the finite population size, the sample size was adjusted using the formula:

n adjusted=1+N(n0−1)n0

where N represents the total population. This adjustment resulted in a minimum required sample size of 377 respondents. For simplicity and statistical robustness, a final sample size of 380 was used, ensuring representativeness of the healthcare workforce in Almaty with a 95% confidence level and a 5% margin of error.

Based on this we have also modified the sample size information in the revised manuscript and it reads as:

*“The sample size of 380 was determined using standard statistical methods for population proportion. Initially, the unadjusted calculation yielded a required sample size of 384 for an infinite population. This was then adjusted for the finite population of 13,267 healthcare professionals in Almaty, resulting in a minimum required sample size of 377. To ensure robust representation, a final target of 380 participants was set, maintaining a 95% confidence level and a 5% margin of error.”*

• (Line 120 – 124/line 130-135) Upon my review of this paragraph, it appears that the study utilizes a sequential mixed methods design; however, it does not specify which type of sequential design has been employed. It is important to note that there are two types of sequential mixed methods designs: exploratory and explanatory. Authors should first define the appropriate design relevant to the study and clarify its application within the research context.

Thabk you for the comment. We used an explanatory sequential mixed methods design. The first phase involved a quantitative survey, followed by a qualitative phase to provide deeper insights that could explain and elaborate on the findings from the quantitative phase. The explanatory nature of the study design is now specified in line 134, and the full description reads:

*"A mixed-methods research design consisting of quantitative surveys and qualitative in-depth interviews was applied in this study to construct a better understanding of HIV-related stigma in primary healthcare. Quantitative survey data were treated as the ‘primary’ data, while qualitative data were collected to complement the survey findings. Thus, the quantitative and qualitative studies were conducted in phases and in a sequential explanatory manner, as described in Fig. 1."*

• (Line 126/line 136) the following subheading should be well formatted “Fig 1. Data collection steps”

Thank you for your comment. We have revised the subheading as suggested. The new title for the figure is now *"Fig. 1 Data Collection Process: Sequential Integration of Quantitative and Qualitative Methods"****.***

• (Line 127/line 137-139 under data collection) This statement is very vague and therefore warrants clarification “The data collection process of this study is described elsewhere”

Thank you for your feedback. We have now clarified this sentence and provided further details in the following paragraph.

*‘The data collection process for this study involved contacting chief medical officers of eight randomly selected polyclinics in Almaty to recruit healthcare providers for a cross-sectional survey, with further details provided in our earlier study [20].’*

• (Line 140/line 149) Describe the structure of the self-administered survey questionnaire that was employed.

Thank you for your comment. The structure of the self-administered survey questionnaire is discussed in the Measures section on line 171 of the manuscript. We have provided a detailed description of the survey's components and how it was designed to assess the relevant variables. We hope this clarifies the structure of the questionnaire as requested.

• (Line 157-158/line 161-170 under measures) Authors must state exactly where readers should look for the relevant information regarding re-validation process of the questionnaires.

Thank you for your feedback. We have clarified the location of the information regarding the re-validation process of the questionnaires in the revised manuscript. The relevant details can now be found in the following statement:

*“The study data were collected using anonymous self-report questionnaires for quantitative The revalidation process, including translation and administration in both languages, is comprehensively outlined in our previously published manuscript on the revalidation of a standardized HIV-related stigma assessment tool in healthcare settings in Almaty [22].”*

• (Line 148 -183/ line 162-170) The article should clearly describe the preexisting scales incorporated into the study for measuring key variables if the questionnaire was not originally developed by authors. It must report the documented reliability coefficients from previous studies and state the internal consistency coefficient obtained in the current study. Additionally, each measure’s items should be outlined by specifying that responses were rated on a Likert scale, including the number of points used for the ratings.

Thank you for your comments. We have added the requested information regarding the internal consistency in the revised manuscript. The updated statement now reads as follows:

*"* *The original assessment tool, validated in six countries (China, Dominica, Egypt, Kenya, Puerto Rico, and St. Christopher & Nevis), was employed to measure stigma through participants' opinions about PLHIV. The tool demonstrated strong reliability in the original validation, with a Cronbach's alpha of 0.78 [21]. For the current study, we revalidated the tool in Kazakh and Russian, achieving excellent internal consistency, with a Cronbach’s alpha of 0.86, indicating its high reliability for the study context. The revalidation process, including translation and administration in both languages, is comprehensively outlined in our previously published manuscript on the revalidation of a standardized HIV-related stigma assessment tool in healthcare settings in Almaty [22]."*

We also clarified in the revised manuscript the information about the response options to Stigma Items on line 180.

*“The primary study outcome was based "Stigmatizing opinions about PLHIV," measured using a 4-point Likert scale with response options ranging from “Strongly Disagree” to “Strongly Agree””.*

• (Line 184 -191/line 225-235) It is beneficial to include a figure that illustrates semi-structured interview guide questions.
Thank you for your suggestion. We agree that including the semi-structured interview guide questions would give more clarity. In response, we will add the interview guide as a supplementary document to the revised manuscript.

• (Line 199/line 218) This statement must be cited “previous research findings.”
Thank you for your recommendation. We have now cited the relevant source.

• (Line 200 -201/line 219-222) “We identified these responses as missing following 201 the questionnaire guide, which led to a decreased sample size to 272 complete responses” Authors should specify their approach to managing missing data, detailing the criteria or thresholds used to determine which incomplete questionnaires were excluded from the analysis. For example, questionnaires with more than 50% of missing data for each participant were excluded from the analysis.
Thank you for your feedback. For the "Fear of HIV" variable, the original tool specifies that "not applicable" responses should be treated as missing. In our data, we observed a significant number of "not applicable" responses, resulting in only 272 complete cases for these items. As a result, this variable was excluded from the regression models, and we have noted this exclusion in the limitations section. We have addressed the issue of missing data in the manuscript as follows on line 219-222 :

*“Fear of HIV items were not included in the regression model due to high numbers of not applicable responses. We identified these responses as missing following the questionnaire guide, which led to a decreased sample size to 272 complete responses. Our descriptive statistics, however, were based on all available data.”*

In terms of missing data more broadly, according to the original scale’s guidelines, participants who answered at least one item from the stigma scale were included in the calculation of the denominator. This approach was followed to minimize missing data in the main analysis, and we have clarified this in the measures section. Thus, aside from the "Fear of HIV" items, we did not encounter significant missing data in our study. We hope this provides clearer clarification on our handling of missing data, particularly in relation to the "Fear of HIV transmission" scale and the overall dataset.

• (Line 213-214/line 232-234) “Qualitative data analysis results were presented using quotations from participants and translated to English by the investigator who is fluent in all three languages.” Authors should clearly explain the translation process, detailing both back-and-forward translation methods to ensure conceptual equivalence. Additionally, they should discuss how the pilot study contributed to refining certain questions for improved clarity and context.

We appreciate the suggestion to clarify the translation process. We analyzed the qualitative data in its original languages (Russian and Kazakh), and the quotations were then translated into English by the primary analyst (BI) for inclusion in this manuscript. To ensure accuracy and conceptual equivalence, back-translation was conducted by two other authors (ZS and EK), who are also fluent in the original languages and English. In response to the feedback, we have revised the relevant section (Lines 229-232) to provide greater clarity, as follows:

*Qualitative data were analyzed in the original languages (Russian and Kazakh), and the primary analyst (BI), who is fluent in all three languages, translated participants' quotations into English. Other research team members (ZS and EK) with fluency in the respective languages reviewed and verified the translated quotes.*

**Results**:
• (Line 228/line 248) Revise this phrase to “…was the most common…” instead of “the commonest..”
Thank you for your feedback. We have made the revision as suggested, changing "the commonest" to "the most common" on line 249.

• (Line 230/line 250) Rephrase “…is homogeneous female dominated.” to “..is predominantly female”
Thank you for the suggestion. We have rephrased the sentence as suggested and it now reads as:

*“ Such gender distribution among the respondents is not surprising since the majority of healthcare workers in the country is predominantly female dominated.”*

• (Line 238/line 259) Remove colon by combining the sentence smoothly “study: 83% (n=380)”
Thank you, we removed it now.

• (Line 257/line 277) “[AOR=0.25, 95%CI (0.09,0.67), p=0.006)” Authors should be consistent between the use of parentheses and square bracket.
Thank you for pointing that out. We have revised the formatting for consistency. The changes made can found in line 275-277.

*“Multivariable logistic regression models which adjusted for the covariates given in Table 2 demonstrated protective effect of longer years of work in healthcare and on having stigmatizing opinions about PLHIV (AOR=0.25; 95%CI=0.09, 0.67; p=0.006) while those have not seen an HIV-positive patient within the last 12 months had higher odds of holding stigmatizing opinions about PLHIV (AOR=3.31; 95%CI =1.73, 6.31; p<0.001).”*

• (Line 277/line 298) Rephrase “medical manipulations” to “medical care or treatment”.
Thank you for your suggestion. We have revised the phrase to: "medical care or treatment"

• (Line 278/line 299) The preferred and non-stigmatising term to refer to this key population is “patients/people living with HIV” instead of “HIV-positive patients”
Thank you for your suggestion. We have corrected the term to "people living with HIV" as recommended.

**Discussion**:

• (Line 364/line 385) Results figures should be included exclusively in the results section and not in the discussion. (“with 87% (n = 380)”)
Thank you for your suggestion. We have now removed this number with corresponding percentage from discussion section and kept it only in the results section.

• (Line 368/line 387) Rephrase “HIV-positive patients” to PLHIV and please address this phrasing throughout the manuscript.
Thank you for your suggestion. We have now rephrased “HIV-positive patients” to “PLHIV” throughout the manuscript for consistency.

• (Line 371-372/line 390-391) Cite those studies immediately “Numerous studies have explored HIV-related stigma in healthcare, primarily focusing on the experiences of PLHIV.”
Thank you for your recommendation. We have now cited the relevant source.

• (Line 406 -407/line 426) This line must be cited “…largely due to widespread homophobia in the country.”
Thank you for your recommendation. We have now cited the relevant source.

• (Line 412/line 429) It is now LGBTQIA+ population and this acronym should be written in full at the first appearance.
Thank you for your suggestion. We have now written the acronym fully o line 430: *gay, bisexual, transgender, queer, intersex, and asexual (LGBTQIA+) individuals*

• (Line 419/line 439) Correct the grammatical error “although there's been….” to “there has been…”
Thank you, corrected now
• (Line 432/line 452) Rephrase “other high HIV prevalence regions…” to “other regions with high HIV prevalence”

Thank you for your suggestion. We have made the requested change and now it reads as:

*“Additionally, the study focused on PHCs in Almaty, limiting generalizability to other regions with high HIV prevalence in Kazakhstan, although findings are consistent with national studies on HIV stigma.”*

• (Line 434/line 454 to “other regions with high HIV prevalence Correct grammar “future research use larger,…” to “future research to uses larger….”
Thank you, we have corrected it now

• (Line 441-442/line 160-170) Authors must report reliability coefficients scores of all measures used to confirm reliability and the process of translation including forward-and back-translation (From English to Kazakh and Russian and from target languages back to the source language) should be also outlined for confirming content validity (Conceptual Equivalence).

Thank you for the comment. Please see the revised version on stigma scales and translation process

“*The study data were collected using anonymous self-report questionnaires for quantitative data and semi-structured interview guides for qualitative data.* *The original assessment tool, validated in six countries (China, Dominica, Egypt, Kenya, Puerto Rico, and St. Christopher & Nevis), was employed to measure stigma through participants' opinions about PLHIV. The tool demonstrated strong reliability in the original validation, with a Cronbach's alpha of 0.78 [21]. For the current study, we revalidated the tool in Kazakh and Russian, achieving excellent internal consistency, with a Cronbach’s alpha of 0.86, indicating its high reliability for the study context. The revalidation process, including translation and administration in both languages, is comprehensively outlined in our previously published manuscript on the revalidation of a standardized HIV-related stigma assessment tool in healthcare settings in Almaty [22].”*

**Conclusions:**

• (Line 446/line 467) This is now new “mid-level medical staff” whereas I initially believed the study was aimed at healthcare providers or professionals in general, without any specific focus on their level of training.
Thank you for pointing that out. We have replaced "mid-level medical staff" with "healthcare staff" for consistency.

• (Line 448/line 471) Rephrase to remain consistent with “HIV care” instead of “AIDS care”
Thank you for your suggestion. We have now made the requested change

• (Line 453/line 477) Rephrase to “combat HIV related stigma” for consistency.
Thank you for your suggestion. We have now made the requested change

**References**:

• (Line 482/line 553) “STIGMA INDEX OF PEOPLE LIVING WITH HIV, 2.0. CAAPL- Central” revise formatting pertaining to the font size accordingly.

Thank you for your suggestion. We have now made the requested change:

*“Central Asian Association of People Living with HIV. People Living with HIV Stigma Index 2.0: Kazakhstan 2022. Almaty: Central Asian Association of People Living with HIV; 2022. Available from:* [*https://www.stigmaindex.org/wp-content/uploads/2022/04/Kazakhstan-SI-2.0-Report-2022_English.pdf*](https://www.stigmaindex.org/wp-content/uploads/2022/04/Kazakhstan-SI-2.0-Report-2022_English.pdf)*”*

Thank you!!

Thank you very much to you!

**Reviewer #2: Overall**

Quantitative data were collected in 2019 (five years ago). It is likely that the HIV epidemic has changed. For example, most countries are nearing epidemic control. Is it possible that the findings are dated? If so, this could be included as a limitation.

We appreciate the observation regarding the timeliness of the data. While global progress towards epidemic control has been notable, Kazakhstan has not yet met the UNAIDS 95-95-95 targets, and little progress has been made in this regard since 2019. Therefore, we did not include this as a limitation. However, in the conclusion, we acknowledged the need for continued research on this topic. The conclusion reads as:

*“While acknowledging recent increases in training frequency since 2019, our study calls for replication studies across primary healthcare centers to track changes over time. We encourage these studies to focus on how stigma has changed over time and its influence on HIV management within the country.”*

Overall, our study reinforces existing research – What exactly is the additional value of this study?

Thank you for your feedback. Along with the finding that those who have had direct contact with PLHIV exhibit lower levels of stigma and the low knowledge of HIV transmission (likely due to limited contact with PLHIV too) this study also provides new insights. It suggests that stigma is not limited to PLHIV but may be even more pronounced towards key populations, such as sex workers and men who have sex with men, as indicated by both qualitative and quantitative data. In response to your comment, we have added the following clarification to the manuscript.

*“The additional value of this study lies in its finding that negative attitudes may not be limited to PLHIV specifically, but also extend to key affected populations, such as sex workers and men who have sex with men.”*

Some stigma studies have recommended a socio-ecological model approach to addressing stigma focusing on the various levels (individual, family/relationship, community, societal). It is unlikely that just focusing on some health-care workers will solve HIV-related stigma. It is likely that in keeping with the socio-ecological model, interventions need to address multiple levels and address intersectional stigma – probably a discussion point. See for example: Ferris France N, Byrne E, Nyamwanza O, Munatsi V, Willis N, Conroy R, et al. Wakakosha "You are Worth it": reported impact of a community-based, peer-led HIV self-stigma intervention to improve self-worth and wellbeing among young people living with HIV in Zimbabwe. Front Public Health. 2023;11:1235150. Epub 20230728. doi: 10.3389/fpubh.2023.1235150

Thank you. We agree with the reviewer’s suggestion and have incorporated the socio-ecological model into the revised manuscript (line 480-483).

*“Additionally, in line with the socio-ecological model, interventions should address multiple levels of stigma, including individual, family/relationship, community, and societal, while also considering intersectional stigma to create a more comprehensive approach [40].”*

**Abstract**
Methods section should say when study was conducted.

Thank you for your suggestion. We have now added the study dates in the abstract as follows: "The cross-sectional surveys took place at the PHCs between May 2, 2019, and July 2, 2019."

Internalized stigma – Terminology is shifting to ‘internal stigma’.

Thank you for your suggestion. We have changed “internalised stigma” to “internal stigma” now on line 85-87

Throughout, would put point (full stop after references i.e. [1,2].

Thank you for your suggestion. We have now made the requested correction

Despite advances made in the clinical management of HIV, 24 % [24%]
do not have an access [do not have access]?
Thank you for your suggestion. We have now made the requested correction

*“Despite advances made in the clinical management of HIV, 24 % of people living with HIV (PLHIV) worldwide do not have access to antiretroviral therapy (ART)…”*

**Methods**
Interviews were conducted until data saturation was achieved. Was the sample of 10 predetermined or guided by the need to achieve theme saturation? It is unlikely that theme saturation could have been achieved with just 10 interviews.

Thank you for the question. The qualitative data collection was driven by the goal of achieving thematic saturation, informed by the key findings from the quantitative data. While 10 interviews provided valuable insights, we acknowledge that additional interviews could have further deepened our understanding. However, due to time and budget constraints, as the study was part of the first author’s PhD research, we were limited in the number of interviews. We now specified this in “Qualitative results” section, line 286-289.

*“Analysis of the qualitative data revealed that we were able to reach data saturation around the key findings from the quantitative data. The list of themes on opinions about PLHIV included fears over HIV transmission, empathetic feelings towards PLHIV and negative feelings towards SWs and people with non-traditional sexual orientations or PWNSO (Fig 4).”*

The questionnaires were self-administered to provide more privacy to the respondents. Were these paper-based or electronic e.g. ACASI, CASI?

Thank you for your question. We have clarified in the manuscript that the questionnaires were paper-based (line 151).

*“The paper-based questionnaires were self-administered to provide more privacy to the respondents.”*

they can comfortably participate in the interviews [could]?

Thank you. Yes, participants were able to comfortably engage in the interviews, which is why the surveys were self-administered with clear instructions provided beforehand. This clarification has now been added to the manuscript.

The questionnaire has several sections that include [had…included]

Thank you for your suggestion. We have now made the requested correction (line 171)

*“The questionnaire had several sections that included socio-demographic information, opinions about PLHIV and fear of contracting HIV.”*

non-clinical-administrative stuff [staff]

Thank you for your suggestion. We have now made the requested correction on line 174
people who agree with at least [agreed]

Thank you for your suggestion. We have now made the requested correction line 182

number of healthcare staff who answered at least of these statements [least ?? of]

Thank you for noticing this. We have corrected it now to “at least one of these statements” on line 188

who have some level of stigmatizing opinion about PLHIV and those who do not have it [who had…who did not]

Thank you for your suggestion. We have now made the requested correction on lines 190 and 191

*“Subsequently, a new binary outcome variable was created for further analysis including those who had some level of stigmatizing opinion about PLHIV and those who did not have it.”*

Sample items on this scale include [included]

Thank you for your suggestion. We have now made the requested correction on line 195

those who do not [did not]

Thank you for your comment. The "Not applicable" option was provided for those who do not engage in medical care or treatment in general. This clarification has now been added on line 199.

*“Response options included a scale of answers from “not worried” to “very worried’. “Not applicable” option was available for those who generally do not engage in medical manipulations.”*

those who have not answered [had not]

Thank you for your suggestion. We have now made the requested correction on line 202

Line 159-160/line 277 and Table 2 knowledge on HIV transmission [of]?

Thank you for noticing this. We have now made the requested correction from “knowledge on HIV transmission” to “knowledge of HIV transmission” both in the line 178 and in the Table 2

For the qualitative data collection, a semi-structured interview guide was used consisting of 20 open-ended questions and guided probes with clarifications used throughout interviews. Please include as supplementary file.

Thank you for your suggestion. The semi-structured interview guide will be included as a supplementary file along with the revised manuscript.

reaching the word count of 15325 words – could leave out?

We agreed and removed this information in the revised manuscript

bilingual nature of the interviews and its [their]

Thank you. We have now made the requested correction on line 227

Each interview was coded by a primary analyst and verified by a secondary analyst. Include initials if they are authors.

Thank you. We have now added initials of the analysts (lıne 231-234)

*“Qualitative data were analyzed in the original languages (Russian and Kazakh), and the primary analyst (BI), who is fluent in all three languages, translated participants' quotations into English. Other research team members (ZS and EK) with fluency in the respective languages reviewed and verified the translated quotes.”*

before the participation [before participating]

Thank you. We have now made the requested correction on line 242

As shown in Table 3, the qualitative interview participants were women – There is nothing in Table 3 that shows they were women.

Thank you for noticing this. We paraphrased this part to avoid confusion. The revised description reads as (line 282-284):

*“All qualitative interview participants were women (n = 10). The respondents' ages ranged from 23 to 60 years, as presented in Table 3. Notably, only two of the ten respondents reported having prior experience working with PLHIV.”*

Separately, Table 3 could leave out the “Speciality” column as this likely identifies the participants e.g. One could guess who the young Endocrinologist is?

We appreciate the concern regarding potential participant identification. However, we believe the data presented are not identifiable for several reasons. Almaty, with a population exceeding 2 million, had over 65 primary healthcare centres at the time of the study, a number that has since increased to 77. We did not disclose the names or specific numbers of the polyclinics, and there is also a high turnover of staff within these facilities. Considering these factors, it is highly unlikely that individual participants can be identified based solely on the information provided in Table 3.

Analysis of the qualitative analysis??

Thank you for pointing that out. We have corrected the typo, and the phrase now reads as "Analysis of the qualitative data" onn line 286.

The majority of the participants…See also Many interviewees mentioned, Most respondents – could include actual numbers. Also, these are only 10 participants who can’t be many even if all?

Thank you for your comment. We have now revised the manuscript to include actual numbers for clarity and transparency. The changes made can be found on line 292 *“All of the participants reported adequate numbers of..”;* line 297 “*Six out of ten interviewees mentioned that having fear of HIV..”;* line 316 “*None of the respondents opposed the idea of PLHIV having children,..”* and line 320 *“Seven out of ten respondents acknowledged drug addiction as a condition that..”*

proposed on more [proposed more]?

Thank you. We have now made the requested correction to “proposed more” on line 294

These concerns have led [led]

Thank you. We have now made the requested correction on line 315

drug addiction as a disease [condition]?

Thank you. We have now made the requested change from “disease” to “condition” on line 318

deep south USA [US]

Thank you. We have now made the requested correction on line 395

during focus group discussions (FGDs) – It does not look like these are described anywhere else? Methods describe only qualitative interviews conducted remotely using social media platforms such as WhatsApp, Zoom and telephone calls

Thank you for your feedback. We would like to clarify that the focus group discussions (FGDs) were part of the revalidation process, which is described in our previously published work on the revalidation of the brief standardized HIV stigma assessment tool in primary healthcare settings in Almaty. We have cited this previous study in the methods section. We have now revised this section to avoid confusion as following:

*“Moreover, during the focus group discussions on the adaptation of the stigma assessment tool, there was a notable lack of awareness regarding MSM, prompting the research team to clarify study terms [22].”*

Additionally, we have added the relevant citation to the previous validation paper, which provides detailed information about the adaptation process and the results of the FGDs.

LGBT – provide full form first mentioned Terminology has shifted to LGBTQI+

Thank you. We have now made the requested correction on line 429:

*“According to one source, the local media often portrays s lesbian, gay, bisexual, transgender, queer, intersex, and asexual (LGBTQIA+) individuals in a negative..”*

It's important, it’s plausible [It is], there's been [there has]

Thank you. We have now made the requested correction on line 436 and 439

Could present study strengths before limitations

Thank you for your comment. However, in our manuscript, we preferred to address limitations first to provide a transparent discussion of the study’s limitations before highlighting its contributions. We believe this sequence allows readers to better appreciate the strengths of the study in context.

future research use larger [uses]

Thank you. We have now made the requested correction on line 451
