# Supplementary material for: “I think they are infected because of their ignorance and lack of responsibility”: A mixed-methods study on HIV-related stigma in the healthcare system in Kazakhstan
Source: PLoS One. 2025 Sep 2;20(9):e0331201. doi: 10.1371/journal.pone.0331201 (PMC12404490; doi:10.1371/journal.pone.0331201)
Supplement: Attachment — Submitted filename: response to reviewers.docx [file pone.0331201.s005.docx]

Manuscript PONE-D-24-31521

Response to Reviewers

Dear Reviewers and the Editorial Team,

We would like to thank you for your time and effort in reviewing our manuscript titled *"I think they are infected because of their ignorance and lack of responsibility": A Mixed-Methods Study on HIV-Related Stigma in the Healthcare System in Kazakhstan*.

In response to your suggestions and comments, we have made the necessary revisions and ensured that all edits are clearly marked for your convenience. Specifically, we have used the review mode with track changes in the manuscript document and highlighted all amendments in yellow in the response letter. In the current document, we also provided our responses in blue font along with updated line numbers for ease of reference.

Once again, thank you for your time, dedication, and expertise. We highly value your contribution to this work and look forward to any further comments you may have.

Sincerely,

Corresponding author.

Journal Requirements:

Please review your reference list to ensure that it is complete and correct. If you have cited papers that have been retracted, please include the rationale for doing so in the manuscript text, or remove these references and replace them with relevant current references. Any changes to the reference list should be mentioned in the rebuttal letter that accompanies your revised manuscript. If you need to cite a retracted article, indicate the article’s retracted status in the References list and also include a citation and full reference for the retraction notice.

Thank you for your feedback regarding the reference list. We have carefully reviewed and corrected the duplicated citations in our manuscript. Specifically:

1. Citation **UNAIDS. Kazakhstan [Internet]** was cited twice (as references 1 and 8). We have replaced the first citation with:

- Obeagu EI, Obeagu GU. A Review of knowledge, attitudes and socio-demographic factors associated with non-adherence to antiretroviral therapy among people living with HIV/AIDS. Int. J. Adv. Res. Biol. Sci. 2023;10(9):135-42.

2. Citation **Davlidova S, Haley-Johnson Z, Nyhan K, Farooq A, Vermund SH, Ali S. Prevalence of HIV, HCV and HBV in Central Asia and the Caucasus: A systematic review** was cited twice (as references 3 and 6). We have deleted the duplicate citation and replaced it with the corresponding source that was missing in our previous submission.

Additionally, we have verified that none of the references in our updated list includes retracted papers.

Additional Editor Comments:

This is an important study about stigma among primary healthcare workers. Although the data were collected some 4-5 years, I believe that the study findings are still relevant to the HIV response globally. I have a few comments in addition to those raised by the reviewers:

1. The authors indicate that for the qualitative component, they interviewed 10 participants were chosen "... from the main sample" for in-depth interviews. It is not clear how and when the 10 participants were selected. Were the 10 participants also interviewed quantitatively? If yes, did the selection happen immediately after the quantitative interview had been completed? If they were interviewed after the quantitative interview, were they informed that they would be invited for a follow-on qualitative interview? In general, the authors should explain: a) how and when the selection of the 10 participants was made, and b) why the selection was restricted to only 10 participants.

Thank you for your important comments. We have addressed your questions regarding the selection of participants for the qualitative component of the study as follows:

a) **How and when the selection of the 10 participants was made?**
 All participants in the quantitative component were informed during the survey process that they might be invited to participate in a follow-up qualitative interview. To maintain confidentiality, we collected phone or work contact numbers from survey respondents for the interview phase. Using a purposive sampling strategy, the 10 participants selected for the qualitative interviews were chosen from these contacts, which included a mix of nurses, doctors, and non-clinical staff such as psychologists and social workers.

The cross-sectional surveys were conducted at the primary healthcare centres (PHCs) in Almaty from May 2, 2019, to July 2, 2019. The qualitative interviews, however, were conducted later, remotely, using platforms like WhatsApp, Zoom, and telephone calls due to COVID-19 restrictions in October 2021. This gap between quantitative and qualitative phases was also due to the primary investigator’s academic commitments abroad (non-certificate degree in the USA as a part of her PhD) during that period. After the investigator’s return to Almaty and after extending the ethical approval for the qualitative phase, the selected participants were invited for the follow-up interviews. We have clarified this timeline and the process in the manuscript.

Specifically, we have revised lines 146-150 and 156 to include this explanation, ensuring the process is clear:

- Line 146-148: *"For the qualitative phase, 10 participants were selected from the survey sample for the in-depth interviews. Using a purposive sampling strategy, we selected a mix of clinical and non-clinical personnel, including nurses, doctors, psychologists, and social workers.”*
- Line 149-150: *"The cross-sectional surveys were conducted at the PHCs in Almaty from May 2, 2019, to July 2, 2019."*
- Line 156-158: *"Due to COVID-19 restrictions, the qualitative interviews were conducted remotely using social media platforms such as WhatsApp, Zoom, and telephone calls due to COVID-19 in October, 2021."*

**b) Why the selection was restricted to only 10 participants?**
The selection of 10 participants was based on the principle of thematic saturation, as guided by the key findings from the quantitative data. The qualitative phase was intended to supplement the larger quantitative phase and therefore more limited in its scope. We have been sure to present themes for which we achieved data saturation among the 10 participant sample. We have specified this reasoning in the "Qualitative Results" section of the manuscript (lines 288-291), where we explain:

*" Analysis of the qualitative data revealed that we were able to reach data saturation around the key findings from the quantitative data. The list of themes on opinions about PLHIV included fears over HIV transmission, empathetic feelings towards PLHIV and negative feelings towards SWs and people with non-traditional sexual orientations (PWNSO) (Fig 4).”*

We hope this clarification resolves your concerns.

2. The authors use the term "participants" to refer to those who participated in the quantitative and qualitative interviews. This can be confusing. I suggest that they use 'respondents' for the quantitative interviews, and 'participants' for the qualitative interviews.

Thank you for your suggestion. We have followed your recommendation and made the necessary changes in the manuscript. We have now used "respondents" to refer to those who participated in the quantitative surveys and "participants" for the qualitative interviews to avoid any confusion.

3. It wasn't clear to me if the 10 participants were key informants or in-depth interview participants. While the rest of the paper refers to "in-depth interviews" - on page 11, the authors refer to these participants as "informants". So, were they interviewed as key informants or in-depth interview participants?

Thank you for pointing this out. To clarify, the 10 individuals were indeed qualitative interview participants, not key informants. We have revised the manuscript to consistently refer to them as "participants" throughout the manuscript, including on page 11, to avoid any confusion.

4. The estimated sample size was 380 but the authors report that they interviewed 448 respondents. No explanation is given for this sort of protocol deviation. Did they seek an amendment to increase the sample size from 380 to 448? Why and how did they end up interviewing more than the estimated sample size? What was the rationale for this? The authors should provide a clear explanation/justification for going over their estimated sample size by up to 68 respondents.

Thank you for pointing out this. Given the positive response to the invitations to the surveys, we decided to include all participants who met the eligibility criteria within the data collection timeframe. This decision was made to ensure inclusivity, maintain a representative sample, and utilize the additional data to enhance the robustness of our findings, i.e. higher statistical accuracy and power. Additionally, we wanted to allow for potential nonresponse, which could affect the representativeness of the sample. Allowing for larger sample size would also allow for higher statistical accuracy and power.

5. Regarding the HIV stigma measurements, can the authors reference any standard tool that they used to collect data? I would like to imagine that stigma validated tools exist and there are standard questions that are used to measure stigmatizing attitudes about HIV, including those used in demographic and health surveys, stigma index, etc. Where did the authors obtain their questions to measure the stigmatizing opinions about HIV?

Thank you for your question. The tool we used to assess HIV stigma is a brief, standardized assessment that has been validated across six countries (China, Dominica, Egypt, Kenya, Puerto Rico, and St. Christopher & Nevis). We referenced the following study for the original validation of this tool:

Nyblade L, Jain A, Benkirane M, Li L, Lohiniva AL, McLean R, Turan JM, Varas‐Díaz N, Cintrón‐Bou F, Guan J, Kwena Z. A brief, standardized tool for measuring HIV-related stigma among health facility staff: results of field-testing in China, Dominica, Egypt, Kenya, Puerto Rico, and St. Christopher & Nevis. Journal of the International AIDS Society. 2013 Nov;16:18718. [21]

Additionally, we revalidated the tool for use in Kazakhstan, specifically in Kazakh and Russian, through translation, factor analysis, and focus group discussions (FGD) in our earlier publication. This process was detailed in a previous publication, which we have cited as reference [20].

We have now specified that we used the standardised stigma assessment tool in the methods section (Lines 162-171):

*“The study data were collected using anonymous self-report questionnaires for quantitative data and semi-structured interview guides for qualitative data.* *The original standardized assessment tool, validated in six countries (China, Dominica, Egypt, Kenya, Puerto Rico, and St. Christopher & Nevis), was employed to measure stigma through respondents’ opinions about PLHIV. The tool demonstrated strong reliability in the original validation, with a Cronbach's alpha of 0.78 [21]. For the current study, we revalidated the tool in Kazakh and Russian, achieving excellent internal consistency, with a Cronbach’s alpha of 0.86, indicating its high reliability for the study context. The revalidation process, including translation and administration in both languages, is comprehensively outlined in our previously published manuscript on the revalidation of a standardized HIV-related stigma assessment tool in healthcare settings in Almaty [20].”*

"Stigmatizing opinions about PLHIV" was measured using the same standardized and re-validated tool on assessing stigma in healthcare based on 5 items which is explained in the following section if the manuscript. Lines 181-190:

*“The primary study outcome was based on "Stigmatizing opinions about PLHIV," measured using a 4-point Likert scale with response options ranging from “Strongly Disagree” to “Strongly Agree”. The number of people who agreed with at least one of the three stigmatizing statements such as “Most people living with HIV do not care if they infect other people”, “People living with HIV should feel ashamed of themselves”, “People get infected with HIV because they engage in irresponsible behaviours” and disagree with the fourth statement “Women living with HIV should be allowed to have babies if they wish” were considered to have some level of stigmatizing opinion about PLHIV [22]. These responses served as a numerator for the stigmatizing opinion calculation while the denominator was based on the number of healthcare staff who answered at least one of these statements [22].”*

6. To what extent are the HIV stigma measurements consistent with other measures of HIV stigma in other settings/countries?

**In terms of the measurement tool:**

As mentioned above, we used an HIV-related stigma scale that had been used on a multitude of other countries. We also validated the tool for use in Kazakhstan before we used it and have referenced an earlier publication that describes this process. Our findings are consistent with research in other settings that have looked at HIV-related stigma in the healthcare setting. We have added the following text to address this comment:

Lines 163-168. *“The original standardized assessment tool, validated in six countries (China, Dominica, Egypt, Kenya, Puerto Rico, and St. Christopher & Nevis), was employed to measure stigma through respondents’ opinions about PLHIV. The tool demonstrated strong reliability in the original validation, with a Cronbach's alpha of 0.78 [21]. For the current study, we revalidated the tool in Kazakh and Russian, achieving excellent internal consistency, with a Cronbach’s alpha of 0.86, indicating its high reliability for the study context.”*

**In terms of the findings, we have also added the following:**
Lines 402-410. *“Numerous studies have explored HIV-related stigma in healthcare, primarily focusing on the experiences of PLHIV [6, 15-18]. However, fewer studies have systematically investigated this issue among healthcare workers, including specific clinic staff such as physicians, nurses, and office managers. Despite this, existing literature consistently indicates moderate to high levels of stigma towards PLHIV within healthcare settings [13, 17, 18]. For instance, a study conducted among healthcare providers in Alabama and Mississippi of the United States (US) found comparable percentages of stigma, with 89% of respondents at urban healthcare centers and 91% at rural clinics demonstrating at least one stigmatizing attitude [25]. Similar findings have been observed in other countries such as Nigeria, China, Poland, and Iran [26-31].”*

Thank you again for all the valuable comments and questions!

**Comments to the Author**

Reviewer #1
Comment 1:

(Line 394-395/lines 406-409) “For instance, a study conducted among healthcare providers in the Deep South US found comparable percentages of stigma, with 89% of respondents at urban healthcare centers and 91% at rural clinics demonstrating at least one stigmatizing attitude [25].”
Specify the context in which the study was conducted. Change “Deep South US” to “among healthcare staff in Alabama and Mississippi of the United States (US)”.

Thank you for your suggestion. We have made the requested change to specify the context of the study. The revised text now reads as follows. Lines 406-409:

*"For instance, a study conducted among healthcare providers in Alabama and Mississippi of the United States (US) found comparable percentages of stigma, with 89% of respondents at urban healthcare centers and 91% at rural clinics demonstrating at least one stigmatizing attitude [24]."*

Comment 2:

(Line 387) Rephrase “stigmatizing opinions about PLHIV” to Stigmatizing attitudes towards PLHIV.
Thank you. We have rephrased it now on lines 387-389:

*“Those who reported experience working with PLHIV within the last 12 months and those with longer years of work in healthcare in general were less likely to hold stigmatizing attitudes towards PLHIV”*

Comment 3:

Throughout the discussion section, it would be clearer and more reader-friendly if the either authors first state the key results or findings of the current study before delving into a comparison with previous studies. Presenting the results upfront ensures the readers understand the main contributions of the study before they evaluate how these findings relate to existing literature. This approach helps to avoids confusion and strengthens the narrative flow of the discussion without missing the bigger picture. Paragraph from line 417 to 426 is an exception to the above comment.

We appreciate this suggestion regarding the structure of the discussion section. In response, we have revised the discussion section to ensure that the main findings are now presented in the opening paragraphs (lines 384-401) before moving on to a comparison with existing literature. This revision indeed had helped to better understand the study findings, thank you.

Comment 4:

In the discussion paragraphs (from line 419 to 449/429-444), the authors addressed discriminatory and stigmatizing incidents against key populations, including MSM, LGBTQIA+, and sex workers. It is imperative to clarify whether these vulnerable groups are also part of the PLHIV community, experiencing compounded stigmatization and discrimination, particularly in primary healthcare settings when seeking HIV care. Otherwise, the stigma discussed may not be directly related to HIV as the primary focal point of the current study.

Thank you. We agree with your point, and our intention was to highlight the general negative attitudes towards MSM, LGBTQIA+, and sex workers. However, following your suggestion, we have now incorporated another source that demonstrates how these groups, with and without HIV, equally face discrimination and human rights violations, particularly in healthcare settings. This is based on a study conducted in several Central Asian countries, which further underscores the compounded stigmatization and discrimination these vulnerable groups’ experiences. Lines 429-444.

*“Widespread homophobia and lack of awareness likely contribute to the heightened HIV-related stigma observed in our study, particularly the strong negative attitudes towards MSM, sex workers, and individuals perceived as engaging in same-sex behaviour. Despite the decriminalization of same-sex relationships, Kazakhstan still faces a pervasive climate of intense homophobia. According to one source, the local media often portray lesbian, gay, bisexual, transgender, queer, intersex, and asexual (LGBTQIA+) individuals in a negative light, leading to increased violence and hostility towards them in public spaces such as parks and nightclubs [32]. These vulnerable groups, including some from HIV communities, seem to experience the most discrimination in healthcare settings [6,18].* *A 2022 study on human rights violations among LGBTQIA+ individuals, with and without HIV, found that Kazakhstan and Kyrgyzstan had some of the highest rates of healthcare-related violations among EECA countries [32]. In particular, Kazakhstan reported the disclosure of HIV medical data in 10% of cases, a figure notably higher than the average (1.1%) observed across the countries studied. It is also important to note that research on attitudes towards LGBTQIA+ people, including our study, primarily focuses on major cities with higher levels of education. Therefore, it is plausible that other regions in the country may exhibit even higher rates of homophobia and stigma towards HIV and key populations.”*

Kovtun O, Tilek kyzy E, Masiumova N. Being yourself is a defect: analysis of documented rights violations related to sexual orientation, gender identity and HIV in 2022 using the REAct system in six eastern European, Caucasus and Central Asian countries. Journal of the International AIDS Society. 2024 Jul;27:e26311

**Reviewer #2:**

Most of my comments have been addressed. I only have a few more, outlined below.

• Stigma towards individuals engaged in same-sex behavior or drug use appeared to be stronger than towards PLHIV overall. Unsure how respondents were able to make the distinction. What if those in same-sex behavior or drug use were also LHIV? Just wondering if the issue of double-stigma emerged or should be explored in future studies – that is both same-sex behavior or drug use and LHIV. See for example: the “double stigma and double trouble” reported by <https://doi.org/10.1371/journal.pgph.0002442>

We appreciate this point. In our quantitative survey, we assessed willingness to provide care to key affected populations with statements such as: “If I had a choice, I would prefer not to provide services to people who inject illegal drugs” (Fig. 1). However, we did not explicitly specify whether these individuals were living with HIV.

Then, in our qualitative interviews, we posed general questions about stigma towards PLHIV as well as towards key affected populations separately. A notable trend emerged while there was relatively greater empathy toward PLHIV in general, when it came to key affected populations, respondents often expressed strong opposition to the behaviours themselves—whether or not the individuals were living with HIV. This suggested that the primary driver of stigma in these cases might stigmatizing attitudes toward same-sex behaviour and drug use rather than solely HIV status.

We thank the reviewing for the citation about “double stigma and double trouble” and made reference to this idea in the Conclusion section as an area to continue to explore in further research. Lines 476-478.

*“The additional value of this study lies in its finding that negative attitudes may not be limited to PLHIV specifically, but also extend to key affected populations, such as sex workers and men who have sex with men. While our research appears to align with the notion of “double stigma and double trouble” [39], this is an area to explore in further research in Kazakhstan.”*

• …around 82% of PLHIV in the country are aware of their status, 68% are on ART, and only around 78% have suppressed viral loads [5].
These figures are confusing as one would expect a cascade - with first figure being highest, second being higher and third being lower than second.

Thank you for your feedback. We agree that the original phrasing of these figures could lead to confusion. To ensure clarity and accuracy, we have revised the sentence to follow the standard HIV treatment cascade framework. The updated version now reads (lines 65-67):

*"Following the Joint United Nations Program on ending AIDS, among PLHIV in Kazakhstan, 82% are aware of their status, 68% of those diagnosed are on ART, and 78% of those receiving ART have achieved viral suppression [5]."*

• Although Kazakhstan has made progress, it has not yet achieved the 90-90-90 targets.
Include a citation of the 90-90-90 targets and the more ambitious 95-95-95 goals by 2025.

Thank you. We have now added the sources on lines 64-67

*“Although Kazakhstan has made progress, it has not yet achieved the 90-90-90 targets [5]. "Following the Joint United Nations Program on ending AIDS, among PLHIV in Kazakhstan, 82% are aware of their status, 68% of those diagnosed are on ART, and 78% of those receiving ART have achieved viral suppression [5]."*

• Although some healthcare providers in resource-limited settings may prefer to visibly mark the files PLHIV for perceived safety reasons, this practice can lead to breaches of patient confidentiality and reinforce stigma [15]. Include [of]: Although some healthcare providers in resource-limited settings may prefer to visibly mark the files of PLHIV for perceived safety reasons…

Corrected. The revised version looks now as following on lines 99-101

*”Although some healthcare providers in resource-limited settings may prefer to visibly mark the files of PLHIV for perceived safety reasons, this practice can lead to breaches of patient confidentiality and reinforce stigma [15].”*

• were restricted to small sample size studies and/or focusing – change to [focused]

Corrected. The revised version looks now as following on lines 111-113

*“Earlier studies conducted on HIV-related stigma in healthcare settings in Kazakhstan were restricted to small sample size studies and/or focused on patients’ perspectives only on addressing this issue [6, 18, 19]”*

• leading to stigmatization of PLHIV using a mixed methods approach. Elsewhere, spelling includes hyphen: mixed-methods

Thank you for the comment. We have now included hyphen in all the spellings of “mixed-methods” throughout the manuscript now.

• There are 65 PHCs in Almaty located evenly among 8 districts. 65/8 may not be evenly distributed?

We acknowledge the concern regarding the distribution of PHCs across districts. Given that the number of PHCs in 2019 was not perfectly proportional to the population size in each district, we employed simple random sampling. To ensure representativeness, we verified whether the selected PHCs covered all districts after the sampling method. Our analysis revealed that only one district –Zhetisu- was not represented. To address this, we conducted an additional analysis to assess potential bias:

First, we compared the average population size of the 7 included districts to the overall average across all 8 districts. The sample average population size was 241,226 which is close to the overall district average of 237,820 indicating that the selected districts are broadly representative in terms of population.

Second, we reviewed the distribution of PHCs across districts. The excluded district had the lowest number of PHCs among all, meaning its absence is unlikely to significantly impact the representativeness of our sample. Given these considerations, while simple random sampling may not guarantee perfect proportionality, our selection remains roughly representative of the broader PHC distribution and population across Almaty.

• Several primary healthcare clinics (n=8) were recruited for the quantitative component based on a simple random sampling technique. Could say: Eight primary healthcare clinics were recruited for…

We have corrected this following your suggestion. Lines 122-123.

*“Eight primary healthcare clinics were recruited for the quantitative component based on a simple random sampling technique”*

• where they can comfortably – change to [could] comfortably

Thank you, “can” is now changed to “could” on line 159.

• The primary study outcome was based "Stigmatizing opinions about PLHIV," measured using a 4-point Likert scale with response options ranging from “Strongly Disagree” to “Strongly Agree”.The – Is its based [on]? Also check space between sentences.

We have now corrected this sentence to: *The primary study outcome was based on "Stigmatizing opinions about PLHIV," measured using a 4-point Likert scale with response options ranging from “Strongly Disagree” to “Strongly Agree”.*

We have checked for the spaces as well, thank you for noticing.

• For the qualitative data collection, a semi-structured interview guide was used consisting of 20 open-ended questions and guided probes with clarifications used throughout interviews (see S1 File) – include reference to the supplement file as suggested.

Thank you. We included the file name now. Lines 205-207.

*For the qualitative data collection, a semi-structured interview guide was used consisting of 20 open-ended questions and guided probes with clarifications used throughout interviews (see S1. Qualitative Interview Guide).*

• which were also utilized to explain variations – see additional spaces

We have checked for the spaces as well, thank you for noticing.

• Written informed consent forms were obtained from the respondents before participating in both quantitative and qualitative arms of the study - Written informed consent was obtained from the respondents before participating in both quantitative and qualitative arms of the study.

Thank you for the suggestion. Following this comment, we have now revised this part. Lines 244-245.

*“Written informed consent was obtained from the respondents before participating in both quantitative and qualitative arms of the study.”*

• Such gender distribution among the respondents is not surprising since the majority of healthcare workers in the country is predominantly female dominated – delete dominated: is predominantly female.

Thank you. The revised version reads as:

*“Such gender distribution among the respondents is not surprising since the majority of healthcare workers in the country is predominantly female.”*

• 14% (n=63) – check space

Thank you. We have now checked for the spaces and corrected where needed.

• Fig 2 demonstrates descriptive statistics of HIV related stigma [HIV-related]

Thank you. The revised version reads as:

*“Fig 2 demonstrates descriptive statistics of HIV-related stigma variables.”*

• around a half of the respondents were aware about the undetectable viral load [around half…were aware of…]

*Thank you. We have revised this part on lines 262-264:*

*“Roughly one third of the respondents correctly answered all the HIV transmission questions (30%, n=129) and around half of the respondents were aware about the undetectable viral load 53% (n= 212).”*

“with non-traditional sexual orientations or PWNSO (Fig 4) – give full form of PWNSO here.

Thank you. We have now given full explanation for the abbreviation on lines 289-291

*“The list of themes on opinions about PLHIV included fears over HIV transmission, empathetic feelings towards PLHIV and negative feelings towards SWs and people with non-traditional sexual orientations (PWNSO) (Fig 4).”*

• In other words, only one quarter… [For example, only a quarter…]

Thank you. We have revised this part on lines 386-387

*“For example, only a quarter of the respondents did not agree with any of the stigmatizing statements about PLHIV in the questionnaire.”*

• one’s HIV status without a consent [one’s HIV status without consent]

Thank you. We have revised this part on lines 413-415

*“The main manifestations of stigma reported in the Stigma Index 2020 included disclosure of one’s HIV status without consent, recommendations on not having sex, gossip among medical workers and avoidance [18].”*

• In our study, we observed differential treatment of PLHIV based on [perceived infection mode].

Thank you. We have revised this part on lines 392-393

*“ In our study, we observed differential treatment of PLHIV based on perceived infection mode. Stigma towards individuals engaged in same-sex behavior or drug use appeared to be stronger than towards PLHIV overall.”*

• According to one source, the local media often portrays s lesbian [delete extra “s” after portrays

Thank you. We have revised this part on lines 432-434

*“According to one source, the local media often portray lesbian, gay, bisexual, transgender, queer, intersex, and asexual (LGBTQIA+) individuals in a negative light, leading to increased violence and hostility towards them in public spaces such as parks and nightclubs [31].”*

• Nonetheless, [to our knowledge], this is the first study to address HIV-related stigma in healthcare using both quantitative and qualitative methods in Kazakhstan.

Thank you. We have revised this part on lines 464-466

*“Nonetheless, to our knowledge, this is the first study to address HIV-related stigma in healthcare using both quantitative and qualitative methods in Kazakhstan.”*
• Effective strategies to combat HIV related stigma [HIV-related]

Thank you. We have now corrected this on line 486
• Acknowledgement [Acknowledgements]

Thank you. We have now revised Acknowledgment to a plural version.
