# Supplementary material for: “I think they are infected because of their ignorance and lack of responsibility”: A mixed-methods study on HIV-related stigma in the healthcare system in Kazakhstan
Source: PLoS One. 2025 Sep 2;20(9):e0331201. doi: 10.1371/journal.pone.0331201 (PMC12404490; doi:10.1371/journal.pone.0331201)
Supplement: Attachment — Submitted filename: response_to_reviewers_auresp_3.docx [file pone.0331201.s006.docx]

Manuscript PONE-D-24-31521

Response to Reviewers

Dear Reviewers and the Editorial Team,

We would like to thank you for your time and effort in reviewing our manuscript titled *"I think they are infected because of their ignorance and lack of responsibility": A Mixed-Methods Study on HIV-Related Stigma in the Healthcare System in Kazakhstan*.

In response to your suggestions and comments, we have made the necessary revisions and ensured that all edits are marked for your convenience. Specifically, we have submitted two versions of the revised manuscript: one with tracked changes and one clean version without them. In the current document, our responses are provided in blue font along with updated line numbers for ease of reference.

Once again, thank you for your time, dedication, and expertise.

Sincerely,

Corresponding author,

Balnur Iskakova, PhD

School of Public Health

Asfendiyarov Kazakh National Medical University

**Additional Editor Comments:**

 The explanation about collecting data from more respondents than the estimated sample size needs to be further tightened. Yes, the researchers' argument that a big sample size improves the accuracy of the estimates and the study power. However, this does not justify interviewing more people than the estimated sample size. A note should be included in the "Discussion" section to state the implications of shooting over and above the estimated sample size and how this might be avoided in future studies.

Thank you for pointing this out. Following your suggestion, we have added this issue on lines 484-489:

*It is also important to mention that although the estimated sample size for this study was 380, 448 eligible respondents were ultimately included. This was due to a high response rate within the data collection period. All eligible respondents were included to improve statistical power and representativeness. However, we acknowledge this as a deviation from the original protocol and recommend that future studies define stopping criteria in advance to manage such scenarios.*

**Review Comments to the Author**

**Reviewer #1:**

 Line 44: Rephrase “HIV-positive” to patients living with HIV, to avoid using stigmatizing statements that refer to PLHIV.

We agree with this point since terminology is important and evolving rapidly. Therefore, we have replaced all instances of *“HIV-positive patients”* with *“Patients living with HIV”* throughout the manuscript to use person-first, non-stigmatizing language.

Line 130: “Mixed methods” is an approach, then 134 “sequential explanatory” is a research design. This should be clearly articulated.

Thank you for this comment. We have now clarified the distinction between the general approach and the specific design, and revised the text accordingly as follows on lines 131-137:

“*A mixed-methods approach was used in this study to construct a better understanding of HIV-related stigma in primary healthcare. Specifically, we employed a* ***sequential explanatory design,*** *in which quantitative survey data were collected and analyzed first as the primary data source. Subsequently, qualitative in-depth interviews were conducted to complement and explain the survey findings. Lastly, we combined the findings from both phases in order to draw conclusions, focusing on how the qualiative data could better explain our quantative results. This design is illustrated in Fig 1.”*

Line 329 -330/lines 340-341: Italicize the verbatim account of a Midwife for consistency.

Thank you for your observation. We have now italicized the verbatim quote for consistency. The revised text appears as follows:

“They are the victims of those who sell drugs for money. It is a profit to someone else. I feel bad for them.” *Midwife, 60 years old*

A sample of 10 participants who participated in the qualitative phase were selected purposefully from those who had already completed a quantitative survey? Or they were not at all part of the first phase. If they were not part of the first phase a rational is needed to elucidate why a different sample was invited. However, if they were part, authors must explain in detail how they were recruited and selected for the second phase.

Thank you for the question. We understand the need for clarity regarding recruiting participants for the qualitative phase. As previously mentioned in the manuscript:

*“For the qualitative phase, 10 participants were selected from the survey sample for the in-depth interviews. Using a purposive sampling strategy, we selected a mix of clinical and non-clinical personnel, including nurses, doctors, psychologists, and social workers.”*

To further clarify, we have now expanded the description of the recruitment process in the Methods section of the manuscript. The following text has been added on lines 151-157:

*"During the quantitative survey, participants were asked if they were willing to be contacted for a potential follow-up in-depth interview. Respondents who agreed to this provided a phone number for further contact. All identifying information such as full names or addresses was not collected to ensure confidentiality. From this pool of respondents who had consented to be re-contacted, 10 participants representing diverse clinical and non-clinical roles were purposively selected and invited to participate in one-on-one interviews at a time convenient for them."*

I have never seen the section that integrate quantitative results with qualitative findings, provided that this study was designed sequentially whereby qualitative findings had to explain quantitative results. Without such integration, this defeats the purpose of sequential explanatory design, as the essence of mixed methods is about combining insights. Therefore, some form of integration is essential.

Thank you for this comment. We agree that the integration of quantitative and qualitative findings is essential in a sequential explanatory design. To address this, we have added a dedicated paragraph in the **Discussion** section to explicitly integrate the findings. The revised section reads as follows on lines 401-415:

*“These findings were further explored in the qualitative component of our study, which confirmed and contextualized these patterns. While the survey revealed widespread endorsement of negative beliefs toward PLHIV and key affected populations, the interviews clarified the moral and cultural judgments underlying these views. Although many participants expressed empathy for the psychosocial challenges faced by PLHIV, this empathy was often conditional and did not extend to those perceived as leading “immoral” lifestyles. Sex workers and MSM were frequently described in explicitly stigmatizing terms, suggesting that stigma is driven not only by fear of infection but also by entrenched moral biases. Furthermore, fear of occupational exposure, reported by the majority of survey participants, was a recurring theme in interviews. Despite acknowledging the availability of protective measures, many participants described this fear as “natural” and emphasized the need to take extra precautions when treating PLHIV. This reflects an underlying anxiety that may contribute to differential or discriminatory clinical practices. The interviews also revealed limited direct contact with PLHIV, and that participants expressed a willingness to engage in more interactive and experience-based training to address HIV-related stigma and discrimination.”*

**Reviewer #2:** I have a few edits:
For the qualitative phase, 10 participants were selected from the survey sample for the in-depth interviews. [delete the - For the qualitative phase, 10 participants were selected from the survey sample for in-depth interviews.]

Thank you. We corrected it now as follows:

*“For the qualitative phase, 10 participants were selected from the survey sample for in-depth interviews.”*

The paper-based questionnaires were self-administered to provide more privacy to the respondents. [Paper-based questionnaires were self-administered to provide more privacy to the respondents.]

Thank you. We corrected it now as follows:

“*Paper-based questionnaires were self-administered to provide more privacy to the respondents.”*

and disagree with the fourth statement [disagreed]

Thank you. We corrected it to the past tense now:

*“The number of people who agreed with at least one of the three stigmatizing statements such as “Most people living with HIV do not care if they infect other people”, “People living with HIV should feel ashamed of themselves”, “People get infected with HIV because they engage in irresponsible behaviors” and* ***disagreed*** *with the fourth statement “Women living with HIV should be allowed to have babies if they wish” were considered to have some level of stigmatizing opinion about PLHIV [22].”*

• then depending on new finding [findings]

Thank you. We have corrected it now to a plural version of the word “finding” on linew 234-236

*“The interviews were coded following the section “Opinions about PLHIV” first deductively then depending on new* ***findings****, inductive codes were added.”*

• demonstrated protective effect [demonstrated a protective effect]

Thank you. We corrected it now as follows on lines 286-287:

*“Multivariable logistic regression models which adjusted for the covariates given in Table 2 demonstrated* ***a*** *protective effect…”*

• while those have not seen [while those had…]

Thank you, this part is corrected now on line 288

*“while those who had not seen a patient with HIV within the last 12 months had higher odds of holding stigmatizing opinions about PLHIV (AOR=3.31; 95% CI =1.73, 6.35; p=0.001)”*

• The qualitative interview participants frequently expressed concerns about the transmission of HIV when they think of …[thought of]

We corrected this now on lines 317-318

*“The qualitative interview participants frequently expressed concerns about the transmission of HIV when they* ***thought*** *of PLHIV, emphasizing the importance of preventive measures.”*

• be careful in choosing sexual partners with females [be careful in choosing female sexual partners]

Thank you, this part is corrected now on lines 354-355

*“According to the data, participants had more negative attitudes towards females, saying that men should be careful in choosing female sexual partners.”*

• They reported feeling of “discomfort” and “disgust” [They reported feelings…]

Thank you, this part is corrected now on line 367

*“They reported feelings of “discomfort” and “disgust” leading to unwillingness to be in contact with such individuals.”*
